# Sexual risk classes among youth experiencing homelessness: Relation to childhood adversities, current mental symptoms, substance use, and HIV testing

**DOI:** 10.1371/journal.pone.0227331

**Published:** 2020-01-03

**Authors:** Diane Santa Maria, Saumali S. Daundasekara, Daphne C. Hernandez, Wei Zhang, Sarah C. Narendorf

**Affiliations:** 1 Department of Research, Cizik School of Nursing, University of Texas Health Science Center at Houston, Houston, TX, United States of America; 2 Department of Health and Human Performance, University of Houston, Houston, TX, United States of America; 3 Department of Biostatistics and Data Science, School of Public Health, University of Texas Health Science Center at Houston, Houston, TX, United States of America; 4 University of Houston, Graduate College of Social Work, Houston, TX, United States of America; Hunter College, UNITED STATES

## Abstract

The aim of this study was to determine whether there are meaningful subgroups with different types of sexual risk behaviors among youth experiencing homelessness and examine the associations between potential classes and other risk variables. A latent class analysis was used to identify classes of youth according to sexual risk behaviors and sexual assault. A two-class solution was found to be the best fit for the data–Lower and Higher Risk groups. The Higher Risk class had significantly higher levels of synthetic marijuana and alcohol use, mental health diagnoses, and were more likely to have been tested for HIV than the Lower Risk group. Youth were more likely to be in the Higher Risk group if they were cisgender female or lesbian, gay, bisexual, or questioning (LGBQ). Nearly all youth (10/11) who reported having HIV infection were in the Higher Risk group. The Lower Risk group were sexually active but had lower rates of risk behaviors and sexual assault. Youth who were not sexually active had the lowest rates of marijuana and alcohol use as well as HIV testing. Health and social service providers should be aware of the added risks for stress, mental distress, mental health diagnoses, and substance use among youth who also report higher risk sexual behaviors and treat as needed.

## Introduction

Youth experiencing homelessness (YEH), ages 14–24 years old, are 6–12 times more likely to become infected with human immunodeficiency virus (HIV) than their housed peers, [1, 2] with HIV prevalence estimates as high as 12% [3]. YEH also have higher prevalence of sexually transmitted infections (STIs) (chlamydia: 2.8–18.3%; gonorrhea: 0.4–24.9%) than the general AYA population (chlamydia: 1.7–3.2%; gonorrhea: 0.3–0.6%) [4, 5] and engage in more sexual risk behaviors than stably housed youth [6, 7]. For example, YEH initiate sex earlier (the median age of sexual debut is 13 years old) [8], are more likely to have multiple partners, to use substances during sex, and are less likely to use a condom [9, 10]. Between 40% and 70% of sexually active YEH report having sex without a condom and 70% of these report condomless sex in the past 3 months [11, 12]. Trading sex is also a survival behavior among YEH that puts them at high risk for HIV/STIs [13]. While rates of trading sex vary among samples of YEH, studies have found that between 10–27% have traded sex to meet their needs while on the streets [14–16]. Many YEH who trade sex do so most frequently for money (82%), a place to stay (48%), or substances (22%) [8, 17, 18].

Youth of color and those who identify as sexual or gender minorities are overrepresented in the homeless population [19]. For example, Black people made up about 13% of the U.S. population in 2018 yet the account for approximately 40% of the homeless population [19]. Similarly, estimates suggest that 29% of YEH identify as LGBQ and about 4% identify as transgender or gender-expansive [20]. YEH who identify as youth of color or LGBTQ frequently encounter violence and face multi-layered stigma, systemic discriminatory, oppressive practices [21–25], and traumas related to racism and cisgenderism [26]. Cisgenderism refers to the structural/systemic oppression trans people face as opposed to the more individual acts of transphobia. These layered and often intersecting factors may put YEH at further sexual risk and risk for sexual exploitation [18, 27].

The literature has strong evidence of the high sexual risks among YEH, yet less is known about whether these risks group together or if there are meaningful groupings within this population who are at higher or lower risk of sexual health sequela. Among urban youth, sexual risks have been found to group together and membership in a high-risk sexual behavior class was associated with substance use [28]. Given that risk behaviors often group together, it is likely that groups identified based on sexual risk levels may also differ on associated risk factors including abuse and trauma, foster care, stress, psychological distress, substance use, mental health, and HIV testing.

### Theoretical framework

This study is guided by the Risk Amplification Model (RAM) [29]. This model demonstrates that adversity, homelessness, and environmental, sociodemographic, and psychosocial factors influence the level of vulnerability experienced by YEH and contribute to risk behaviors, such as condomless sex. RAM has been used to explore the determinants of risk behaviors and negative outcomes among YEH [30–33]. RAM suggests that an approach that considers the combination of risk and vulnerabilities to HIV infection and other STIs among YEH may lead to improved prevention strategies over uniform prevention programs. Classes of risk have been found using latent class analyses (LCA) among HIV positive injective drug users [34]. Using LCA, various risk profiles have also been found for experiencing victimization with differentiated associations of risk of substance use related to those profiles [35]. Differences in sexual risk behaviors have also been found by risk profile using LCA for young adult intravenous drug users [36]. As well, research suggests a co-occurrence of HIV infection and trauma among people living with HIV [37]. Several known risk factors such as adverse childhood experiences, being involved in the foster care system, mental health, and substance use may differentially be associated with classes of sexual risk.

#### Adverse Childhood Experiences (ACEs)

Experiencing early adversities greatly impacts the risk for subsequent homelessness and risk behaviors. In one study among young men who have sex with men, the odds of being unstably housed were greater among those who more frequently experienced lack of basic needs (e.g., food, hygiene, clothing) and physical abuse during childhood [38]. One study found that 47% of YEH reported sexual abuse, 31% left home because they were sexually abused by their parents, 27% left home because they were emotionally abused by their parents, and 20% left home because of parental physical abuse [39]. While having higher ACEs can increase the risk of experiencing homelessness and YEH frequently experience more ACEs, it can also increase engagement in risk behaviors. In a study among 362 young adults, experiencing trauma prior to age 18 was associated with problematic substance use, difficulty discerning or heeding risk, and self-destructiveness [40].

#### Foster care

The literature strongly suggests that involvement in the foster care system is a risk factor for poor outcomes in young adulthood including experiencing homelessness and sexual risk behaviors [41]. Youth with a history of foster care are more likely to be diagnosed with HIV or an STI, have more frequent condomless sex, and use alcohol or drugs during sex compared to their peers in the general population [42]. Among a sample of YEH, Hudson et. al. (2012), found that a higher proportion of former foster youth reported trading sex compared to youth who had never been in foster care [11]. However, little is known about whether there is an association between foster care and clusters of sexual risk behaviors.

#### Stress

Stress is another contributing factor to engaging in risk behaviors. A growing body of evidence has shown that stress is associated with risky sexual behaviors across various populations including young females and young men who have sex with men. Stress predicts inconsistent use of condoms [43] and moderate to severe stress is linked to increased frequency of sexual intercourse [44]. In African American adolescent females, higher stress was associated with a lower rate of condom use, inconsistent condom use, and not using a condom during their most recent sexual encounter [45]. Among young men who have sex with men, reporting high stress on the day of sex predicted inconsistent condom use [46, 47]. While stress has been associated with sexual risk behaviors, to date no research has examined this relation among YEH. Further research is needed on the role of stress for potential inclusion in prevention models for risk behaviors in this population [48–51].

#### Substance use

Overdose is one of the leading causes of death among YEH [52]. Substance use can be both a cause and a consequence of life on the streets, and a growing body of research attests to higher rates of substance use among YEH [53]. Substance use rates among YEH may be twice that of housed youth [10]. In one study, 86% of YEH 18–25 years old met the DSM-IV diagnostic criteria for a substance use disorder [10] compared to only 15% in the general young adult population. Using substances can also lead to further risk. For example, a recent study found that same day drug use increases the odds of engaging in sexual activity among YEH [54].

#### Psychological symptoms and mental health diagnosis

In a nationally representative sample, approximately 19% of YEH reported being depressed [55], compared with 11% of housed youth [56]. Other studies report rates of 8% [57] to 61% [58] for depression and 5% to 48% [15, 59, 60] for post-traumatic stress (PTS) among YEH. Depression among YEH may be due to a disproportionate burden of lifetime adversity, including abuse, neglect, and housing instability [61]. Stress and depression related to homelessness are likely to be related to classes of sexual risk. For example, depression and negative affect adversely influence HIV risk. Depression is associated with HIV risk behaviors such as injection drug use and condomless sex [43, 62, 63]. In a sample of predominantly African American YEH, Castro et. al., found that a higher number of psychiatric diagnoses was positively correlated with higher number of sexual risk behaviors [64], and improvements in depressive symptoms were positively associated with decreases in the overall HIV risk index and number of sex partners. The literature suggests that depressive symptoms are a risk factor for HIV risk behaviors [62, 65, 66] and that stress, mental health, and substance use should be considered when assessing sexual risk among YEH [66]. The chronic stress of being homeless can cause physiological changes that affect how an individual reacts to his/her environment. Acute stress impairs executive functioning, working memory [67], flexible task-goal implementation [68, 69], and impulse control [70]. Impaired executive functioning leads to poor decision-making ability [71] and lower inhibitory (i.e. impulse) control, which can lead to sexual risk behaviors and substance use [72].

Clearly, the literature suggests that adverse childhood experiences, being involved in the foster care system, mental health, and substance use contribute to sexual risk. Yet, little is known about whether there are classes of sexual risk behaviors among YEH that necessitate different approaches to prevention and whether these classes are correlated to other risks in unique ways. However, classes of sexual risk have been found among other youth populations. Among sexual minority youth, patterns of sexual initiation differ by gender and cluster by sexual act timing characteristics [73] and sexual risk profiles differ among youth who use intravenous drugs [36] Differences in mental health and substance use by sexual risk classes have been found among truant youth with males in a higher sexual risk group experiencing more attention deficit hyperactivity disorder problems and high risk females reporting more marijuana use and depression symptoms [74]. Finally, among foster care youth, which are overrepresented among YEH populations, there are subgroups of youth; those experiencing the highest levels of adversity also reported the highest sexual risk. These finding suggest that there are classes of risk among other high risk youth populations and there are likely classes of higher and lower risk among YEH. These classes may further inform targeted strategies [75].

While YEH engage in sexual risk behaviors at high rates and the literature supports the need to assess stress, mental health symptoms, and substance use, it is less well established whether there are subgroups of YEH that cluster together based on higher or lower levels of sexual risk as indicated by different types of risk behaviors. Further, while several contributing factors to engaging in sexual risk behaviors have been established in the literature including ACEs, foster care, substance use, and having mental health diagnosis, less understood is the role of stress and psychological distress on sexual risk behaviors. Finally, little is known about whether these associations differ based on subgroups of YEH clustered by levels of risk and whether HIV testing differs by risk level.

The aims of this study were 1) to determine if there are meaningful subgroups of youth with different clusters of sexual health risk among YEH and 2) to assess for associations between the subgroups and experiences of adversities, current levels of stress and mental distress, substance use, mental health diagnosis, and HIV testing or infection. Therefore, we used a latent class analysis (LCA) to identify classes of sexual risk among YEH. Latent class analysis is a statistical method that is used to identify hidden subgroups within a population that may vary based on a combination of chosen indicators and is an appropriate approach to explore whether different indicators of sexual risk may cluster together and differentiate youth that belong to different subgroups within the overall population of youth experiencing homelessness. Following identification of the sexual risk latent classes, we examined the demographic characteristics of the subgroups. We then assessed for adverse childhood experiences, foster care, current stress and psychological distress, substance use, mental health diagnoses, and HIV testing and infection across the classes.

## Methods

Participants were recruited over four weeks as part of a methodological study to count and survey YEH in Harris County, Texas, during October and November 2014 [76]. Youth were eligible if they were between the ages of 13 and 24 years and were either homeless or unstably housed. Youth were recruited from shelters, street outreach, magnet events (e.g., hot meals) and drop-in service centers. We conducted preliminary qualitative studies and information gathering sessions with the local homeless coalition, the Police Department Homeless Outreach Team, and the project Community Advisory Group to create a list of locations, days, and times to conduct recruitment. Over the four week recruitment phase of the study, we visited 47 different locations in 97 separate recruitment events.

At each recruitment event, eligibility screening was conducted by trained volunteers (primarily social work and nursing students) for all presenting youth who appeared under age 40. Field notes from all recruiting study staff indicated that approximately 5% of approached youth refused to participate. The most frequent reason for not participating was lack of time to take the survey. If eligible, all youth participants provided verbal consent and took a paper-based or audio-assisted computer survey in a private setting that took approximately 20–30 minutes to complete. The Institutional Review Board approved a waiver of parental/guardian consent for minors who participated in this study. No identifiable information was obtained from participants. To reduce duplication across recruitment sites, the same team members went to the same recruitment sites to facilitate facial recognition, and youth were asked during screening whether they had taken the survey before. Participating youth received a $10 gift card to a local restaurant or grocery store. All study procedures were approved by the University of Texas Health Science Center at Houston, Institutional Review Board and the University of Houston, Committee on the Protection of Human Subjects Review Board according to the principles expressed in the Declaration of Helsinki.

### Measures

#### Demographic characteristics

Youth were asked to self-identify their age, gender identity, sexual orientation, and race/ethnicity. Youth indicated whether they identified as cisgender male, cisgender female, transgender, non-binary gender or something else and those who identified as transgender, non-binary gender or something else were recoded into transgender/non-binary category. Youth could identify as a sexual minority by choosing lesbian, gay, bisexual, or questioning (LGBQ) which was dichotomized into LGBQ or heterosexual. Last, youth could choose multiple categories of race/ethnicity or self-identify as multiracial or other. Those that chose multiple categories were re-coded as multiracial for analysis.

#### Sexual risk factors

Sexual risk items were adapted from the single item indicators used in Youth Risk Behavior Survey [77]. Youth were asked if they had ever had sex. Those who responded “yes” were then asked the age at which they first had sex; if they drank alcohol or used drugs before their most recent sexual encounter; if they had been tested for HIV in the past year; if they had ever had an STI; if they had ever traded sex for money, drugs, or a place to stay; if they had used a condom or other method of contraception the last time they had sex; whether they had more than four lifetime sexual partners; and if they had ever had anal sex. We also inquired about rape as a sexual risk factor by asking participants if they had ever had sex against their will.

#### Homelessness or housing instability

To determine current housing situations, youth were asked during eligibility screening where they had spent the previous night. This variable was collapsed into 3 categories: sheltered (including transitional living), literally homeless (i.e., staying on the streets), or unstably housed (i.e., doubled up or couch surfing). If youth reported staying with a friend or relative the prior night but were unsure of where they could stay in the next 30 days, they were categorized as unstably housed.

#### Childhood adversities

Adverse Childhood Experiences (ACE) were assessed using the ACE scale which asked whether the participant had experienced each of ten traumatic events before the age of 18 [78]. Foster care history was assessed by asking youth if they had ever spent time in foster care.

#### Psychological symptoms and diagnoses

Perceived Stress was measured using the 4-item short-form Perceived Stress Scale (PSS-4) to assess how often respondents found their life situation stressful, unpredictable, and uncontrollable over the previous month using a five-point Likert scale rated from 0 (never) to 4 (very often) [79–81]. We used standard score cutoffs (≥9 points on the PSS-4) to denote moderate/severe stress symptoms for descriptive purposes. This scale has been found to have acceptable validity and reliability (α = .77) in prior studies of psychometric properties [67] but had lower reliability in this sample (α = .57). Psychological distress was measured with the Kessler-6 using a five point Likert scale that assesses the frequency of six different symptoms over the previous month including feeling nervous, hopeless, restless or fidgety, worthless, depressed, or that everything was an effort [82]. The Kessler-6 has a Cronbach’s alpha of 0.78 among YEH in prior studies [83] and had good reliability in this sample (α = .88). We utilized the recommended clinical cutoff of 13 on the scale to create a dichotomous indicator for whether a respondent was positive for psychological distress. Mental Health Diagnosis were assessed by asking youth if they had ever been diagnosed with each of the following diagnoses: ADHD, bipolar, depression, conduct disorder or oppositional defiant disorder, schizophrenia, or post-traumatic stress disorder.

#### Substance use

Substance use was assessed using items adapted from the Monitoring the Future study which asked youth to report whether they had used marijuana, synthetic marijuana, or alcohol in the past 30 days [84].

## Data analysis

Our study used measures in two different ways. First, we used indicators of sexual risk behavior to conduct latent class analysis and identify subgroups with different sexual risk profiles. Variables included in the examination of the sexual risk classes included: used alcohol/drugs before sex, had anal sex, had four or more lifetime sexual partners, used a condom at last sex, had a history of an STI, sexual debut before age 14, reported forced sex, and traded sex.

The association between the assigned class membership and a second set of auxiliary variables were studied after the classes were created according to a three-step LCA approach. The auxiliary variables included demographic characteristics and risk variables. At the first step of LCA, those that had never had sex were not included in classification model as their answers to the indicators of sexual risk were not available. They naturally formed a distinct group with lowest risk. For those that were sexually active, each of the sexual risk indicator variables were utilized to fit a latent class model using MPlus v8.3 [2, 85].

We examined the fit of four models (one to four-class models) assessing three fit indices -the Standardized Bayesian Information Criteria (SBIC), the Bayesian Information Criteria (BIC), and the Akaike Information Criteria (AIC) to select the optimal number of classes. These are three different fit indices that are all used to measure the relative quality of a finite number of models, with the lowest number indicating the best fitting model. For the SBIC, BIC, and AIC, we assessed whether the numbers went down when adding additional classes as smaller numbers indicate better fit. We also used the Vuong Lo Mendell Rubin (VLMR) Likelihood Ratio Test and the Bootstrap Likelihood Ratio Test (BLRT) to test whether a class with one additional class was a significantly better fit than a model with one fewer class. In addition, we examined the composition of the classes identified to ensure that they made conceptual sense and provided meaningful subgroups.

At the second step LCA, the Modal classification method was applied. Modal classification method assigns each individual a class with the highest membership probability [86–88]. Classification errors were calculated according to an Equation (6) of Vermunt’s [89]. This choice of classification method allowed us to include those who had never had sex into the later analysis of the associations between sexual risk and other auxiliary variables.

The third step of LCA was first performed by computing two-way tables summarizing the class membership probabilities per auxiliary variable category [89]. The class membership probabilities of different categories were compared via the Wilcoxon Rank Sum test or Kruskal-Wallis test depending on the number of classes yielded from the previous steps. In addition, the group who had never had sex was included in the post hoc analyses with modal assignment to further examine the relations between the sexual risk classes and the auxiliary demographic and risk variables. Chi-square and ANOVA tests were used to identify significant differences between the sexual risk classes on each of the auxiliary variables. Due to the multiple comparisons conducted, the level of significance was adjusted by considering p < .001 statistically significant. The analyses in the third step were performed in SAS 9.4 and SPSS Statistics v17 (IBM Corp.).

## Results

The sample included 434 youth aged 13–24 years; 212 completed a paper-based survey, and 222 completed an audio-assisted computer survey. Youth with missing values on the sexual risk questions were excluded from the analyses for a total of 416 youth in the current analysis. The sample was primarily 18–24 years old (87%) and African American (54%), and the mean age was 20 years (SD = 2.68). More than half of the sample identified as male (54%) and 22% identified as lesbian, gay, bisexual, or questioning (LGBQ). Half of the sample had spent the previous night in a shelter, 32% were on the streets, and 18% had stayed with an acquaintance, friend, or relative. The sample was predominantly sexually active (n = 320; 77%). Sexual risks were high among sexually active youth with 68% reporting multiple sexual partners, 46% reporting not using a condom at last sex, 34% early sexual debut, and high rates of forced sex (31%) and engaging in trade sex (30%) (Table 1).

**Table 1:** Sexual risk indicators used to create classes and conditional probabilities.

	LCA Sample % (N = 320)	Lower Risk % (N = 229)	Conditional Prob (SE)	Higher Risk % (N = 91)	Conditional Prob (SE)
Anal sex	22.5	11.2	0.11 (0.02)	47.9	0.48 (0.07)
> Four sex partners	68.1	57.5	0.58 (0.04)	91.8	0.92 (0.04)
Used condoms at last sex	53.9	61.4	0.61 (0.04)	36.7	0.37 (0.06)
History of STI	21.5	15.6	0.16 (0.03)	35.1	0.35 (0.05)
Sexual debut before age 14	33.7	32.1	0.32 (0.04)	37.1	0.37 (0.06)
Forced sex	31.2	14.2	0.14 (0.03)	69.7	0.70 (0.07)
Trade sex	30.4	5.9	0.06 (0.03)	84.5	0.85 (0.08)
Substance use with sex	34.7	28.0	0.28 (0.03)	49.7	0.50 (0.06)

SE = Standard Error

### Latent sexual risk classes

**Fig 1:**
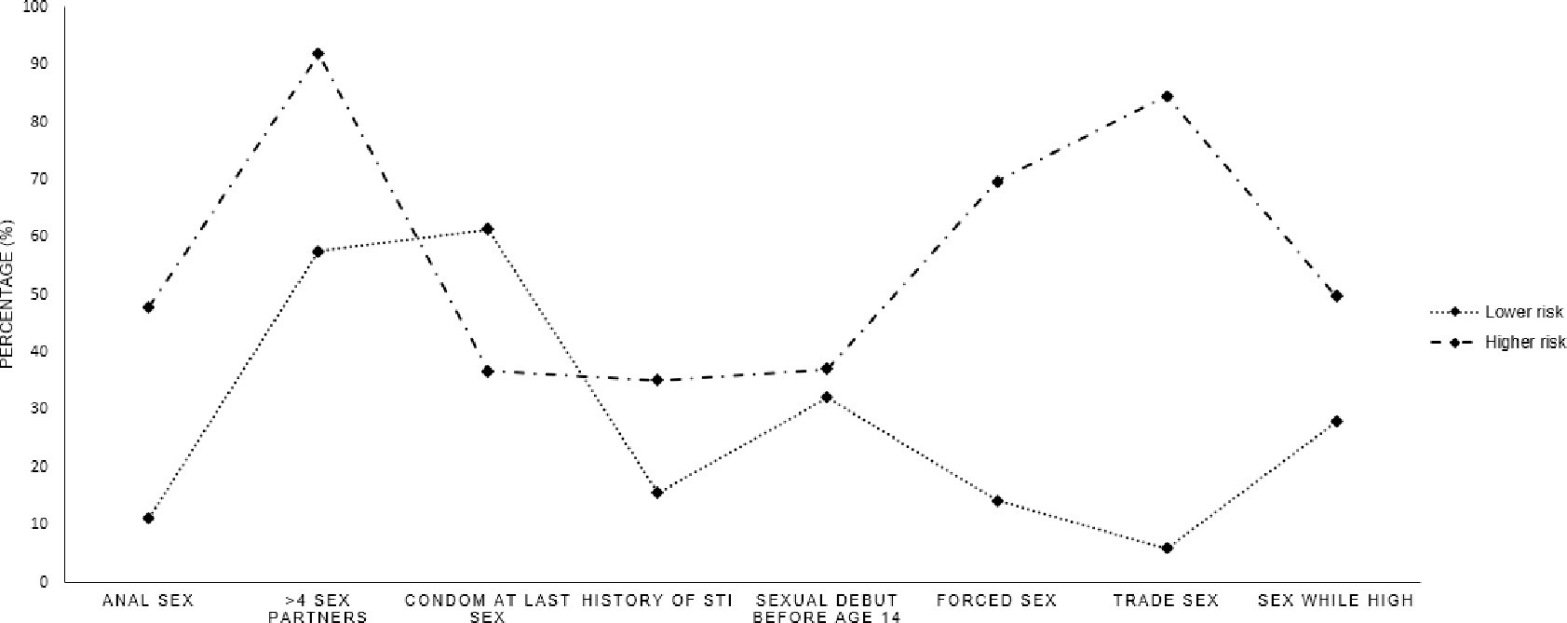
Risk characteristics of sexual risk classes.

A two-class solution was considered the best fitting model (Table 2). All fit indices were lower in the two class solution than the one class solution, indicating better fit, and greater in the three and four class solution, pointing to a two class solution as the best fit. In addition, the significance tests of the VLMR LRT and the BLRT indicated that a two class solution was a significantly better fit than a one class solution but that the three and four class solutions were not better than the two class solution. The results were examined to ensure they made conceptual sense (Fig 1) prior to accepting the final two class solution.

**Table 2:** Fit statistics used to select classes.

	1 Class	2 Class	3 Class	4 Class
AIC	3111.737	2990.104	2993.017	2995.610
BIC	3142.322	3055.096	3092.416	3129.417
SBIC	3116.944	3001.169	3009.940	3018.391
VLMR LRT		137.019; P<0.0000	14.805; P = 0.1825	15.407; P = 0.1288
BLRT		139.633; P<0.0000	15.087; P = 1.000	15.407; P = 1.000

#### Class descriptions

Since there were only two classes, we only calculated and compared the probabilities of the low risk class since the probabilities of the high-risk class were one minus the corresponding probabilities of low risk class. Differences in class characteristics emerged. The largest majority of youth in the sample were Lower Risk (56%) with 22% being Higher Risk. The first class, labelled as “lower risk” included the majority of sexually active participants (n = 229, 72%; see Table 2, Fig 1). The youth in the Lower Risk group reported lower rates of anal sex, having multiple sexual partners, a history of STI, early sexual debut, forced sex, and trade sex than the higher risk group. Youth in the Lower Risk group also had higher rates of condom use at last sex compared to Higher Risk youth. The second class was labeled as “Higher Risk” (n = 91) and was characterized by higher rates of all sexual risk factors than the lower risk group including engaging in trade sex, experiencing forced sex, having an STI, and having multiple sexual partners. Youth in the Higher Risk group also reported lower rates of condom use at last sex. The overall classification error was 0.206. Specifically in our study, the classification errors were:

P(W=2|X=1)=0.038

P(W=1|X=2)=0.168

#### Demographic and risk indicators by sexual risk group

Examination of the sample by sexual risk group included youth that reported they had never had sex as a distinct group compared to the lower sexual risk and higher sexual risk classes identified through the LCA. There were significant differences across the groups in age, gender, sexual orientation and race/ethnicity but no differences in living situation (see Table 3). Results were not different when the probability of class assignment was included in analyses of the classes generated through the latent class analysis. The results of these specific analyses are presented in Table 1.

**Table 3:** Total sample demographics and comparisons across the classes (N = 416).

	Total sample n (%)	Never had sex n (%)	Lower Risk n (%)	Higher Risk n (%)	P Value
Overall Sample	416	96 (23.1)	229 (55.0)	91 (21.9)	
***Demographics***					
Age					<0.001
Young Adult (18–24)	360 (86.5)	71 (74.0) ^b^	204 (89.1)	85 (93.4)	
Minor (<18)	56 (13.5)	25 (26.0) ^b^	25 (10.9)	6 (6.6)	
Gender					<0.001
Cisgender Male	223 (53.5)	62 (64.6)^b^	130 (56.8)	31 (34.1)	
Cisgender Female	176 (42.4)	30 (31.3)^b^	94 (41.0)	52 (57.1)	
Transgender/non-binary	17 (4.2)	4 (4.1)	5 (2.2)^c^	8 (8.8)	
LGBQ	89 (22.4)	21 (22.8)	34 (15.2)^c^	35 (40.7)	<0.001
Race/Ethnicity					0.033
African American	225 (54.2)	55 (57.3)	128 (55.9)	42 (46.7)	
White	54 (13.0)	10 (10.4)	22 (9.6)	22 (24.4)	
Hispanic	47 (11.3)	12 (12.5)	23 (10.0)	12 (13.3)	
Multiracial	67 (16.1)	13 (13.5)	43 (18.8)	11 (12.2)	
Other	22 (5.3)	6 (6.3)	13 (5.7)	3 (3.3)	
Living Situation					0.912
Sheltered	209 (50.2)	49 (51.0)	111 (48.5)	49 (53.9)	
Literally Homeless	132 (31.7)	30 (31.2)	74 (32.3)	28 (30.8)	
Unstably Housed	75 (18.0)	17 (17.7)	44 (19.2)	14 (15.4)	
Childhood Factors					
ACE Score	4.16 (3.1)	2.86 (2.5) ^b^	3.93 (3.1) ^c^	5.97 (2.9)	<0.001
Foster Care Experience	172 (42.0)	33 (34.7)	95 (42.4)	44 (48.4)	0.167
Mental Health Diagnosis					
ADHD	178 (44.3)	37 (39.8)	89 (40.6)	52 (57.8)	0.014
PTSD	96 (24.1)	19 (20.4)	38 (17.6)^c^	39 (43.3)	<0.001
Bipolar	199 (49.8)	36 (38.7) ^b^	101 (46.5) ^c^	62 (68.9)	<0.001
Depression	204 (50.8)	41 (44.1) ^b^	96 (43.8) ^c^	67 (74.4)	<0.001
Conduct/ODD	67 (16.8)	12 (13.0)	33 (15.3)	22 (24.4)	0.080
Schizophrenia	67 (16.8)	13 (14.0)	33 (15.2)	21 (23.3)	0.159
Psychological Symptoms					
Moderate/High Stress	169 (41.6)	32 (33.7)	86 (38.7)	51 (58.0)	0.002
Psychological distress	189 (47.6)	41 (45.1)	97 (44.5)	51 (58.0)	0.088
Substance Use					
Marijuana use	134 (35.8)	17 (19.3) ^a^	82 (40.6)	35 (41.7)	0.012
Synthetic Marijuana	66 (17.9)	9 (10.1) ^b^	30 (15.0) ^c^	27 (33.8)	<0.001
Alcohol	152 (36.5)	15 (15.6) ^a^^,^^b^	87 (38.0)	50 (55.0)	<0.001
Tested for HIV	245 (79.6)	0 (0)	167 (75.9)	78 (88.6)	0.009
HIV positive	11 (3.7%)	0 (0)	1 (0.5)^c^	10 (11.4)	<0.001

SD: standard deviation; df: degrees of freedom; LGBQ: lesbian, gay, bisexual, or questioning; Moderate to Severe Stress: PSS≥ 9; ADHD: Attention Deficit Hyperactivity Disorder; PTSD: Post Traumatic Stress Disorders; ODD: Oppositional Defiant Disorder

^a^ Never had sex group different from Lower risk group, p < 0.001

^b^ Never had sex group different from Higher risk group, p < 0.001

^c^ Lower risk group different from Higher risk group, p < 0.001

Youth that never had sex were more likely to be under 18 years of age compared to high risk classes (p < .001). A higher percentage of cisgender females and LGBQ youth were in the Higher Risk group compared to the other two groups. Nearly all youth who self-reported having HIV infection (10/11) were in the Higher Risk group compared to the Lower Risk group. There was also a greater percentage of youth that identified as transgender or non-binary gender in the Higher Risk group.

The number of different adverse childhood experiences was highest among youth in the Higher Risk group and lowest among youth in the Never Had Sex group. While there was a high rate of foster care experience among the total sample (42%), there were no significant differences across the classes in foster care status. Regarding stress and distress, there was a difference in stress levels among the groups that was approaching significance, with youth in the Higher Risk group reporting the highest rate compared to the Lowest Risk and No Risk groups (58.0%, 38.7%, 33.7%, respectively). There were no significant difference in psychological distress between the groups.

The risk groups were also significantly different in their use of substances. The No Risk group had the lowest rates of marijuana (19%), synthetic marijuana (10%), and alcohol use (16%) compared to the Lower Risk group. The Higher Risk group had the highest rates of substance use. In addition, there were differences among the risk groups in their prior mental health diagnoses. The Higher Risk class had the highest reported rates of all the reported mental health diagnoses among the three groups.

## Discussion

This study adds to the literature on the evidence of classes of high sexual risk among YEH by providing evidence to support that there are subgroups of YEH that have distinctly different levels of sexual risk who may warrant different interventions and that these groups vary by gender identity, sexual orientation, and substance use. The largest subgroup of youth were Lower Risk with another sizable proportion of YEH not engaging in sex, especially minors. A smaller proportion of youth (22%) were in the Higher Risk group. This indicates that among YEH, there is a distinct, yet smaller, subgroup that exhibit high risk sexual behaviors across most risk indicators who also have higher exposure to childhood adversities, more mental health needs, and higher substance use and are disproportionately cisgender female and LGBQ. The findings support the need for healthcare and social services providers to create youth-friendly and affirming environments that consider gender identity, sexual orientation, and level of sexual risk in programs targeting sexual health, mental health, and substance use to meet the needs of these meaningful groupings of YEH.

The highest sexual risk subgroup, which had the highest ACE score and mental health diagnoses was disproportionately cisgender female and LGBQ. This group differed most drastically on engaging in trade sex and experiencing forced sex and the proportion of youth who had multiple sexual partners. Nearly all cases of HIV infection were among youth in the Higher Risk group. Conversely, the Higher Risk group also exhibited some increased protective behaviors compared to the other groups with higher proportions of HIV testing. This may suggest that HIV testing efforts are in fact reaching the highest risk YEH. Due to the increased rates of trade sex and forced sex among Higher Risk youth, programs should consider targeting cisgender females and LGBQ youth regarding sexual assault HIV prevention and post-sexual assault care awareness and access.

The findings from this study support the Risk Amplification Model and suggest that there are grouping of risk; a higher risk group of YEH is characterized by clusters of multiple sexual risk behaviors and substance use and also experience more childhood adversities and mental health diagnoses. These cumulative risks may also be influenced by the moderately to high stress that was reported by the Higher Risk group which, in turn, have been found to increase the risk for engaging in sexual risk behaviors [7, 44, 45, 47]. However, the influence of stress which approached significance may differ by gender. The highest risk group was disproportionately cisgender female. Further a small proportion of the individuals in the study identified as transgender; similar to prior research transgender youth were more likely to be in the high sexual risk group [27]. While measuring structural factors associated with experiencing homelessness is beyond the scope of the current study, experiencing discrimination has been associated with transgender youth being less likely to exit homelessness [90]. The structural factors that prevent transgender youth from exiting could also be placing them at greater jeopardy for engaging in multiple sexual risk behaviors.

Prevention interventions should target sexual victimization among YEH with high risk sexual behaviors; the highest risk group reported higher rates of sexual assault i.e. being forced to have sex against their will. Victims of sexual violence also engage in high risk sexual behaviors [91, 92]. Therefore, interventions that aim to decrease sexual risks should also address the prevention of sexual victimization as well as treatment and recovery. Trauma-informed interventions that integrate treatment and coping strategies should be used to help youth with high risk sexual behaviors and histories of sexual trauma.

Mental health is also associated with classes of sexual risk. Youth with a diagnoses of a mental health disorder were more likely to be in the higher sexual risk group, particularly youth that reported a PTSD, bipolar, or depression diagnosis. This aligns with the literature that suggests that YEH with mental illness also exhibit more sexual risks [66]. The data also suggest the need for trauma-informed approaches to assist YEH with stress management strategies as part of sexual health promotion. As found in the literature, stress may be contributing to sexual risk behaviors [70, 72, 93] with the most highly stressed youth also reporting the most sexual risk factors. Sexual health interventions among YEH must consider the mental health needs and assist youth in accessing mental health services and stress management interventions as a complementary strategy to supporting youth in reducing sexual risks.

Finally, we found that the overall rates of HIV testing in the past year were approaching the CDC Healthy People 2020 recommended levels for the proportion of adolescents and adults who have been tested for HIV in the past 12 months. These findings are similar to other studies among YEH that have found about 82% have been tested for HIV in the past year [27, 94]. HIV testing was highest among the high-risk groups but could be improved among the Low Risk group. While rates of HIV testing met the CDC recommendations of annual testing for the general population, both Higher and Lower Risk YEH may benefit from more frequent HIV testing such as every three to six months due to the high prevalence of condomless sex, trade sex and forced sex among Higher Risk youth.

Several limitations should be considered when interpreting the results of this study. While we used facial recognition and inquiry to reduce the risk for duplication, it is possible that a participant could have taken the survey more than once. With this cross-sectional design, we have established groups of youth by risk level and explored for differences in auxiliary risk variables across the groups. In addition, some measurement problems with our cross sectional approach should be noted. Perceived stress was based on the youth’s experiences in the past month, while most of the sexual risk indicator measures assess lifetime risk (e.g., ever had sex against their will, ever had an STI, ever traded sex, ever had anal sex). While unlikely, it is possible that some of the ‘ever’ sexual behaviors occurred in the past month and did not precede the past month measure of stress. Nevertheless, the association suggests that stress may be important factor to target in sexual health interventions among YEH that needs further exploration.

Regarding sexual risk behaviors measures, it should be noted that the level of risk associated with behaviors such as condomless sex and anal sex depends on various factors such as the individual and their partner’s HIV and STI status, and whether the youth is adherent to PrEP as prevention or HIV treatment for HIV-positive individuals. And, this sample also originated from one geographic location. While large and diverse, it may not be representative of youth in other locations or from rural settings. Measurement challenges with forced sex and sexual debut also exist. Note that we did not ask specifically whether sexual debut was the result of forced sex. Therefore, there is overlap in these variables that should be noted and addressed. Future studies should consider this in designing measures and ask questions that better enable disentangling these specific issues. One way to do this would be to conduct cognitive interviews with youth to determine if they classify sexual debut as the day they were forced to have sex. Finally, we did not capture which modality (e.g. paper or tablet) the participant used to complete the survey. Since this was done at random, we do not believe there would be group differences by modality, though we cannot confirm this.

Despite the limitations, this study provides evidence that classes of sexual risk differ by gender and sexual orientation and that and substance use differ by these clusters of sexual risks. Further research using longitudinal and real-time measures such as ecological momentary assessments to assess the relation between stress, mental health, and substance use is warranted to better understand the role of stress and mental health symptoms on sexual risk and substance use decision making. Given our finding that sexual risk indicators cluster together, targeting any one risk behavior may translate into reduced risk for others.

There are several notable strengths of this study. The sampling methods used and large sample size of a hard-to-reach population of YEH recruited from various shelters, drop-in centers, magnet events, and the streets is an improvement over previous studies in this population that relied on small convenience samples of service-engaged youth. Another strength is the examination of different types of sexual risks assessed in addition to condomless sex, such as substance use-related sex, sex against one’s will, and trading sex, which adds to the discussion of risks that contribute to a high HIV prevalence in YEH. Finally, this analysis demonstrates that in addition to well-established risk indicators, stress may be an additional contributor to sexual risk among YEH. While stress has theoretical underpinnings to risk decision making, less research has examined its relation to sexual risk behaviors among YEH or other youth known to have high levels of stress.

Future studies can improve the exploration of how stress differentially affects sexual risk behaviors in YEH by using a higher validity stress scale. PSS had lower reliability in this sample than found in the literature [67]. Perhaps a more granular measure of stress of the streets to assess different stress sources (e.g., meeting basic needs, strained family relations, victimization on the streets, encounters with the police) would be an improved way to measure stress [95] and determine how it relates to lower and higher sexual risk subgroups. As well, using ecological momentary assessments may advance how we examine the influence of real-time mood and affect, including stress, on sexual risk behaviors [96, 97] that produce the subgroups found in this study. The development and testing of sexual health interventions that target stress and psychological distress as a means of reducing sexual risks may be warranted.

Additional concerns raised by this data are the relatively low use of condoms. Youth often underestimate their risk for HIV even when considered clinically eligible for HIV prophylaxis based on risk behavior assessments [27]. Because this data is cross sectional and cannot assess variations in sexual risk behaviors or one’s reaction to risk perception over time, further research is needed to determine if youth do in fact increase HIV prevention behaviors when perceptions of risk or actual risk are elevated.
